# The effect of mild induced hypothermia on outcomes of patients after cardiac arrest: a systematic review and meta-analysis of randomised controlled trials

**DOI:** 10.1186/s13054-015-1133-0

**Published:** 2015-12-01

**Authors:** Xi Wen Zhang, Jian Feng Xie, Jian Xiao Chen, Ying Zi Huang, Feng Mei Guo, Yi Yang, Hai Bo Qiu

**Affiliations:** Department of Critical Care Medicine, Zhongda Hospital, School of Medicine Southeast University, No.87, Dingjiaqiao Road, Gulou District, Nanjing, 210009 China

## Abstract

**Introduction:**

Mild induced hypothermia (MIH) is believed to reduce mortality and neurological impairment after out-of-hospital cardiac arrest. However, a recently published trial demonstrated that hypothermia at 33 °C did not confer a benefit compared with that of 36 °C. Thus, a systematic review and meta-analysis of randomised controlled trials (RCTs) was made to investigate the impact of MIH compared to controls on the outcomes of adult patients after cardiac arrest.

**Methods:**

We searched the following electronic databases: PubMed/MEDLINE, the Cochrane Library, Embase, the Web of Science, and Elsevier Science (inception to December 2014). RCTs that compared MIH with controls with temperature >34 °C in adult patients after cardiac arrest were retrieved. Two investigators independently selected RCTs and completed an assessment of the quality of the studies. Data were analysed by the methods recommended by the Cochrane Collaboration. Random errors were evaluated with trial sequential analysis.

**Results:**

Six RCTs, including one abstract, were included. The meta-analysis of included trials revealed that MIH did not significantly decrease the mortality at hospital discharge (risk ratio (RR) = 0.92; 95 % confidence interval (CI), 0.82–1.04; *p* = 0.17) or at 6 months or 180 days (RR = 0.94; 95 % CI, 0.73–1.21; *p* = 0.64), but it did reduce the mortality of patients with shockable rhythms at hospital discharge (RR = 0.74; 95 % CI, 0.59–0.92; *p* = 0.008) and at 6 months or 180 days. However, MIH can improve the outcome of neurological function at hospital discharge (RR = 0.80; 95 % CI, 0.64–0.98; *p* = 0.04) especially in those patients with shockable rhythm but not at 6 months or 180 days. Moreover, the incidence of complications in the MIH group was significantly higher than that in the control group. Finally, trial sequential analysis indicated lack of firm evidence for a beneficial effect.

**Conclusion:**

The available RCTs suggest that MIH does not appear to improve the mortality of patients with cardiac arrest while it may have a beneficial effect for patients with shockable rhythms. Although MIH may result in some adverse events, it helped lead to better outcomes regarding neurological function at hospital discharge. Large-scale ongoing trials may provide data better applicable to clinical practice.

**Electronic supplementary material:**

The online version of this article (doi:10.1186/s13054-015-1133-0) contains supplementary material, which is available to authorized users.

## Introduction

Millions of people suffer sudden cardiac arrest (CA) every year in the whole world, often related to coronary heart disease. The global incidence of out-of-hospital cardiac arrest (OHCA) is about 82.9 per 100,000 population within all age groups, and 213.1 per 100,000 population in adult groups [1]. Return of spontaneous circulation (ROSC) is achieved in 25 % to 40 % of the patients [2, 3]. However, the mortality and risk of neurological impairment is high. Global brain ischemia and the reperfusion injury following resuscitation may lead to brain tissue degeneration and loss of neurological function [4].

The 2010 guidelines of the American Heart Association recommend mild induced hypothermia (MIH; 32–34 °C) as an important part of resuscitation for patients who have experienced CA [5]. Two randomised controlled trials (RCTs) have shown the beneficial effects of MIH in the improvement of survival and neurological outcomes of patients following CA [6, 7]. In addition, it has been shown in rats that hypothermia protects brain regions that display rapid as well as delayed neuronal damage and that a minimal time of hypothermia is necessary for effective neuronal protection [8]. However, MIH also interferes with numerous physiological and pathological processes and might induce unfavourable effects, such as cardiac dysrhythmia and coagulopathy [7, 9]. Recently, many scholars have questioned the temperature and the efficacy of MIH and have criticised previous trials that showed that MIH improved the mortality of patients with OHCA because they had high risks of bias [10]. Nielsen and colleagues have shown that in unconscious survivors of OHCA of presumed cardiac origin, hypothermia at a targeted temperature of 33 °C did not confer a benefit compared with a targeted temperature management of 36 °C [11]. Although MIH has been implemented as the standard care for patients after CA in many countries, the evidence for its possible beneficial effects are still controversial. Thus, the object of this systematic review and meta-analysis was to evaluate the impact of MIH compared to controls with temperature >34 °C in adult patients after CA on mortality and neurologic performance as main outcomes.

## Methods

### Eligibility criteria

We included trials with the following features:

1. Type of study: Randomised controlled clinical trials
2. Population: Adult patients (aged more than 18 years) who suffered from CA (regardless whether in-hospital CA (IHCA) or OHCA) and who were successfully resuscitated
3. Intervention: MIH (any body target temperature ≤34 °C)
4. Control (treatment according to the standard treatment after CA in any body temperature >34 °C with or without temperature intervention)
5. The following outcomes were included. a) Primary outcomes: mortality at hospital discharge, mortality at 6 months or 180 days and long-term (more than 1 year); b) secondary outcomes: neurological function during hospital stay and 6 months or 180 days in cerebral performance categories (CPC) and adverse events.

### Search strategy for the identification of studies

We conducted a search of the following databases until December 2014: Medline, Embase, Cochrane (Central) database, Elsevier, Web of Science and ClinicalTrials.gov (inception to December 2014). Searches were conducted as described by Nielsen et al. [10] and Arrich et al. [12] (see Additional file 1: Tables S1 and S2). There was no language restriction.

### Study selection

Two reviewers independently screened titles and abstracts to determine whether a particular study met the inclusion criteria. The full texts of the articles were then reviewed independently according to the inclusion and exclusion criteria. Any discrepancies were resolved by a consensus on the inclusion or exclusion of a particular study after a discussion with a third reviewer.

### Data extraction and management

Two reviewers independently extracted data using a standardised data extraction protocol. Any disagreements between the two reviewers were resolved by a discussion, whereby a consensus was then reached. The relevant outcomes were mortality, neurological function and adverse events potentially related to MIH. Neurological function was evaluated according to CPC, where a CPC score of 1 and 2 was defined as a good neurological outcome and a score of 3–5 was defined as a poor neurological outcome. We further defined outcome at discharge as short-term outcome and outcome at 6 months as long-term outcome.

### Methodological risk of bias assessment

We summarised the evidence applying GRADE levels [13] (high, moderate, low, and very low) by evaluating design, quality, consistency, precision, directness and possible publication bias of the included trials using GRADEpro Guideline Development Tool.

We assessed random sequence generation, allocation concealment, blinding of participants and personnel, blinding of outcome assessment, incomplete outcome data and selective reporting to assess the internal validity of the identified trials according to the Cochrane Handbook [14].

Sensitivity analyses were used to assess the impact of study quality issues on the overall effect estimate and the effect size of all identified trials when neglecting heterogeneity and publication status conducted by STATA 11.0 (Stata Corporation, College Station, TX, USA).

### Subgroup meta-analysis

A subgroup meta-analysis was performed to determine the effect of the initial rhythms (shockable and non-shockable rhythms) on the efficiency of MIH with regard to the outcomes of patients with CA. The articles without specific classification of initial rhythms were classified into shockable and non-shockable rhythm groups.

In all included studies, we also performed meta-analysis to compare the effects between MIH and no target temperature (any body temperature >34 °C without cooling or warming methods) on the outcomes of adult patients after CA. Moreover, meta-analysis of the effects between MIH and target temperature management (TTM; any body temperature >34 °C with cooling or warming methods) on the outcomes of adult patients after CA was also performed.

### Statistical analysis

The meta-analysis of the effect of MIH on outcomes in patients with CA was conducted using the methods recommended by the Cochrane Collaboration software RevMan 5.3 (The Nordic Cochrane Centre, Rigshospitalet, Copenhagen, Denmark). The statistical heterogeneity and inconsistency were measured and quantified by the Mantel-Haenszel (M-H) chi-square test and the I^2^ test in RevMan 5.3 [15]. The statistically significant heterogeneity was predefined as *p* < 0.10 with the M-H chi-square test. In addition, I^2^ index was used to assess heterogeneity in the meta-analysis. Higgins and colleagues proposed 25 %, 50 % and 75 % of I^2^ values would mean low, medium and high heterogeneity, respectively [15]. In cases of obvious heterogeneity (i.e., *p* < 0.10 with M-H test; I^2^ > 50 %), the meta-analysis employed the random-effects model; otherwise, the meta-analysis used the fixed-effects model. We reported a risk ratio (RR) with 95 % confidence interval (CI) for the dichotomous data and weighted mean differences with 95 % CIs for the continuous data. The publication bias was evaluated by visual inspection of the funnel plot.

Due to type I errors which result from an increased risk of random error and repeated significance testing [16, 17], we used trial sequential analysis (TSA; TSA software version 0.9 Beta; Copenhagen Trial Unit, Copenhagen, Denmark), which combines information size estimation with an adjusted threshold for statistical significance in the cumulative meta-analysis [16–18]. Information size was calculated as diversity-adjusted information size (DIS) [19], suggested by the relative risk reduction (RRR) of the intervention in the included trials.

## Results

### Summary of the studies

**Fig. 1:**
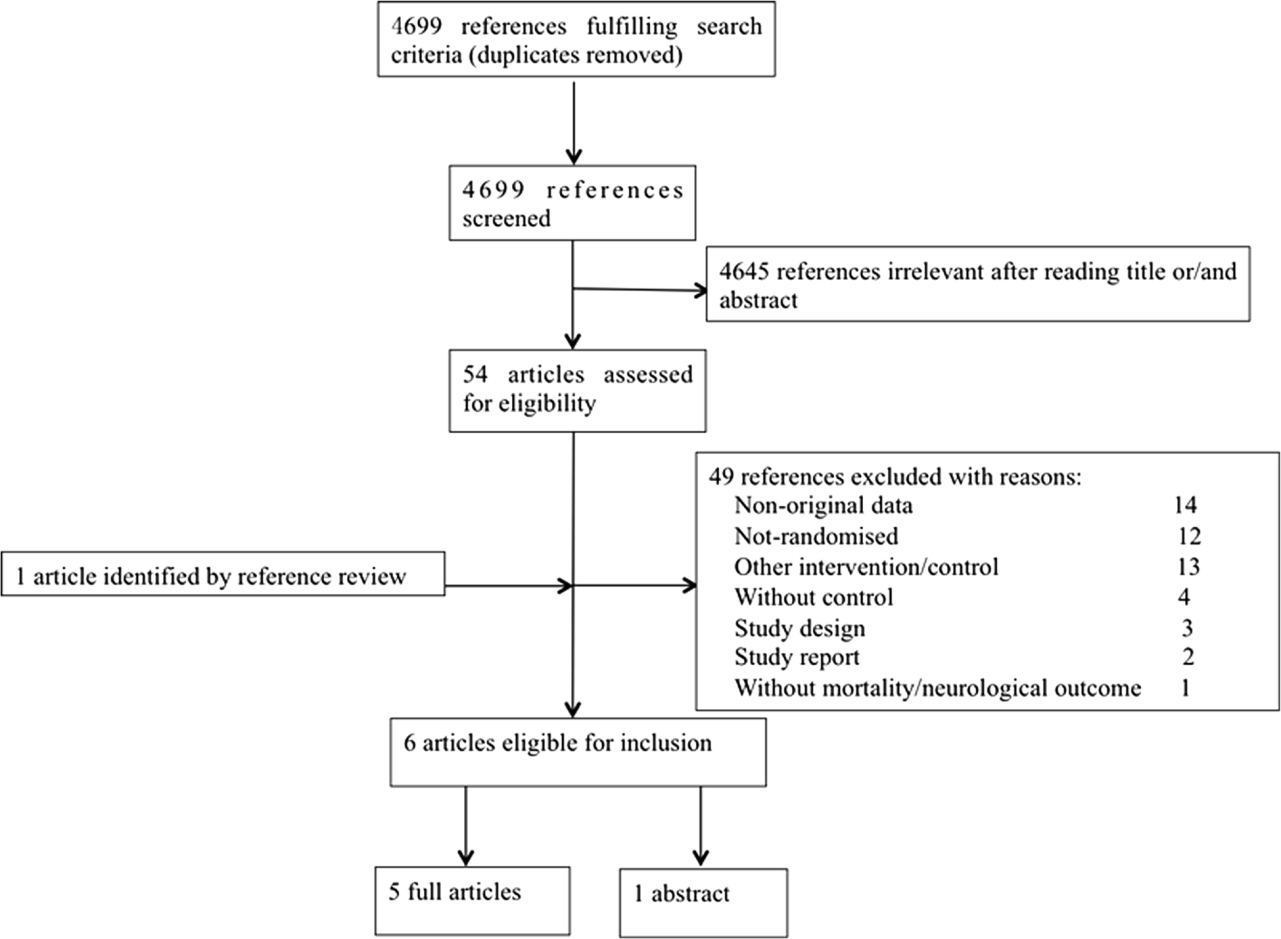
Flow diagram of the study selection

The flow diagram that shows the identification of studies throughout the review is illustrated in Fig. 1. Overall, we identified 4699 papers and excluded 4645 after screening the titles and abstracts for the terms “hypothermia”, “cardiac arrest” and “randomised control trial”. We retrieved 54 articles that were full-length manuscripts and, finally, six were included in this meta-analysis.

These six trials included 1417 patients. The subjects who were included were adult patients with CA who were randomised to MIH of 32–34 °C versus control intervention. A total of 730 subjects received hypothermia therapy while the remainder were in the control group. The methods of hypothermia therapy included external cooling, the use of an intravascular cooling device and continuous renal replacement therapy (CRRT). One study did not report the method of cooling [20]. Among all the included studies, three studies followed the patients for 6 months [7, 11, 21], and the others followed the patients for 14 days [22], 1 month [20] or until hospital discharge [6]. The main characteristics of the included studies are shown in Table 1.

**Table 1:** Characteristics of the included trials

	Duration	Participants	Experimental intervention	Control intervention	Inclusion criteria	Exclusion criteria	Follow-up time	Patients screened (n)	Patients included (n)
Mori 2000 (abstract) [20]	Not reported	OHCA patients with GCS <8	MIH to 32–34 °C for 72 h, method of cooling not described, rewarming rate not reported	36 °C for 72 h, method of temperature control not described	OHCA and GCS <8	Not defined	1 month	Not reported	54
Hachimi-Idrissi 2001 [22]	6 months	Unconscious OHCA patients, cardiac cause of arrest, initial rhythm asystole of PEA	Helmet cooling to 34 °C, when temperature of 34 °C achieved or more than 4 h elapsed from start of cooling, passive rewarming for 8 h	Standard ICU care, acetaminophen if temperature over 38 °C	OHCA of cardiac origin asystole or PEA as initial rhythm, >18 years, temp >30 °C, GCS <7	Pregnancy, coagulopathy, CNS antidepressant medication before CA, cardiogenic shock (MAP <60), GCS ≥7	14 days	Not reported	30
HACA 2002 [7]	65 months	Unconscious CA patients, cardiac cause of arrest, initial rhythm VF or non-perfusing VT	Air cooling induced hypothermia to 33 °C for 24 h, passive rewarming for 8 h	Standard ICU care, no temperature control	Witnessed CA of cardiac origin, VF or non-perfusing VT as initial rhythm, 18–75 years, 5–15 min from arrest to CPR and <60 min to ROSC	<30 °C, coma because of drugs before CA, pregnancy, response to verbal command, MAP <60 for >30 min, hypoxemia >15 min, terminal illness, factors making follow-up unlikely, coagulopathy, other study, CA after arrival of medical personnel	6 months	3551	275
Bernard 2002 [6]	33 months	Unconscious OHCA patients, cardiac cause of arrest, initial rhythm VF or VT	Ice-pack induced hypothermia to 33 °C for 12 h (started prior to hospital admission), active rewarming for 6 h	Standard ICU care, no temperature control	OHCA with VF as initial rhythm, persistent coma	<18 years for men, <50 years for women, cardiogenic shock <90 SBP despite epinephrine, other causes of coma than CA, no available ICU bed	Hospital discharge	Not reported (84 eligible)	77
Laurent 2005 [21]	23 months	Unconscious OHCA patients, cardiac cause of arrest, initial rhythm VF or asystole	CVVH to 32–33 °C (CVVH for 8 h and surface cooling for 16 h), passive rewarming	CVVH maintaining 37 °C for 8 h, thereafter no temperature control	OHCA of cardiac origin, VF of asystole, 18–75 years, <10 min to start of CPR, <50 min to ROSC	Pregnancy, response to verbal command, terminal illness before CA	6 months	244	42
Nielsen 2013 [11]	27 months	OHCA patients with GCS <8	Ice-cold fluids, ice packs, and intravascular or surface temperature-management devices induced hypothermia to 33 °C for 28 h, gradual rewarming to 37 °C in hourly increments of 0.5 °C, <37.5 °C for unconscious patients until 72 hours after CA	Ice-cold fluids, ice packs, and intravascular or surface temperature-management devices induced hypothermia to 36 °C for 28 h, gradual rewarming to 37 °C in hourly increments of 0.5 °C, <37.5 °C for unconscious patients until 72 hours after CA	OHCA of cardiac origin, GCS <8, >18 years, >20 min of spontaneous circulation after resuscitation	An interval from the ROSC to screening >240 min, unwitnessed arrest with asystole as the initial rhythm, suspected or known acute intracranial haemorrhage or stroke, <30 °C	180 days	950	939

*CA* Cardiac arrest, *CNS* Central nervous system, *CPR* Cardiopulmonary resuscitation, *CVVH* Continuous veno-venous filtration, *GCS* Glasgow Coma Score, *HACA* Hypothermia After Cardiac Arrest, *ICU* Intensive care unit, *MAP* Mean arterial pressure, *MIH* Mild induced hypothermia, *OHCA* Out-of-hospital cardiac arrest, *PEA* Pulseless electrical activity, *ROSC* Return of spontaneous circulation, *SBP* Systolic blood pressure, *VF* Ventricular fibrillation, *VT* Ventricular tachycardia

### Random errors

**Fig. 2:**
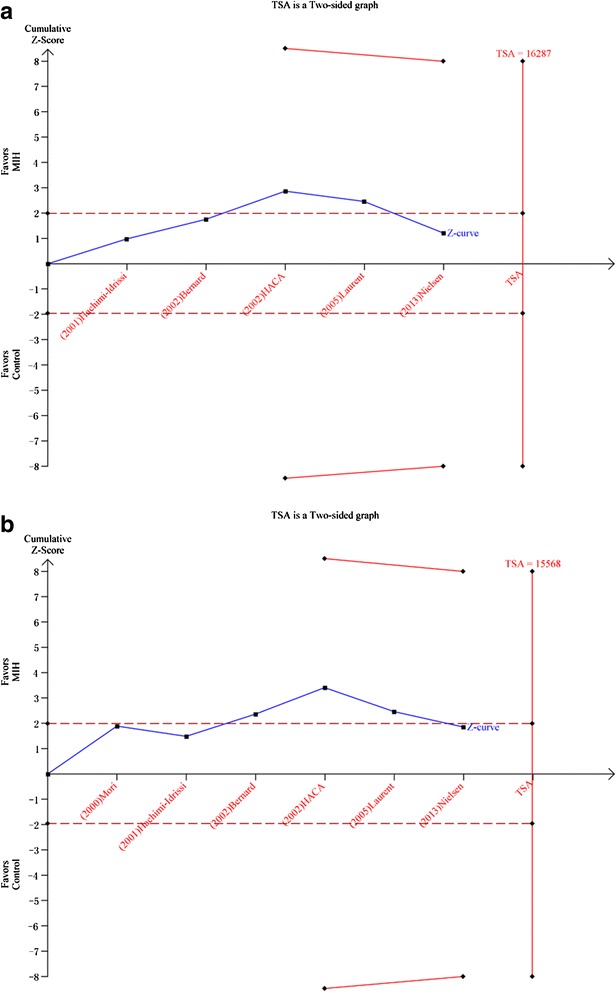
**a**. Trial sequential analysis for a relative risk reduction of all-cause mortality of 5.8 % of hypothermia after cardiac arrest in five trials with 1363 patients reporting mortality. A required diversity-adjusted information size of 16,287 patients was calculated based on a control event proportion of 51.0 %, a hypothermia-induced relative risk reduction of mortality of 5.8 % suggested by all trials, α = 0.05 two-sided, β = 0.20 (power = 80 %), and diversity D^2^ = 60 %. The cumulated Z-curve (*blue*) crosses the traditional boundary (*p* = 0.05) but not the trial sequential monitoring boundary, indicating lack of firm evidence for a beneficial effect of 5.8 % relative risk reduction of the intervention when the analysis is adjusted for repetitive testing on accumulating data. There is insufficient information to reject or detect an intervention effect of 5.8 % relative risk reduction of all-cause mortality as the required information size is not yet reached. **b**. Trial sequential analysis (*TSA*) for a relative risk reduction of 7.29 % of hypothermia after cardiac arrest in six trials with 1409 patients reporting neurological function. A required diversity-adjusted information size of 15,568 patients was calculated based on a control event proportion of 56.9 %, a hypothermia-induced relative risk reduction of poor neurological function of 7.29 % suggested by all trials, α = 0.05 two-sided, β = 0.20 (power = 80 %), and diversity D^2^ = 79 %. The cumulated Z-curve (*blue*) crosses the traditional boundary (*p* = 0.05) but not the trial sequential monitoring boundary, indicating lack of firm evidence for a beneficial effect of 7.29 % relative risk reduction of the intervention when the analysis is adjusted for repetitive testing on accumulating data. There is insufficient information to reject or detect an intervention effect of 7.29 % relative risk reduction of poor neurological outcome as the required information size is not yet reached

TSA was calculated with α = 0.05 and β = 0.20 (power 80 %) and a required diversity-adjusted information size based on the intervention effect suggested by the included trials using a fixed-effects model (RRR of 5.8 % regarding mortality and 16,287 patients) and a random-effects model (RRR of 7.29 % regarding poor neurological function and 15,568 patients). TSA indicated lack of reliable and conclusive evidence for a beneficial effect of MIH for both mortality (Fig. 2a) and a poor neurological outcome (Fig. 2b), since the monitoring boundaries were not finally surpassed and the required information size was not reached.

### Impact on mortality

**Fig. 3:**
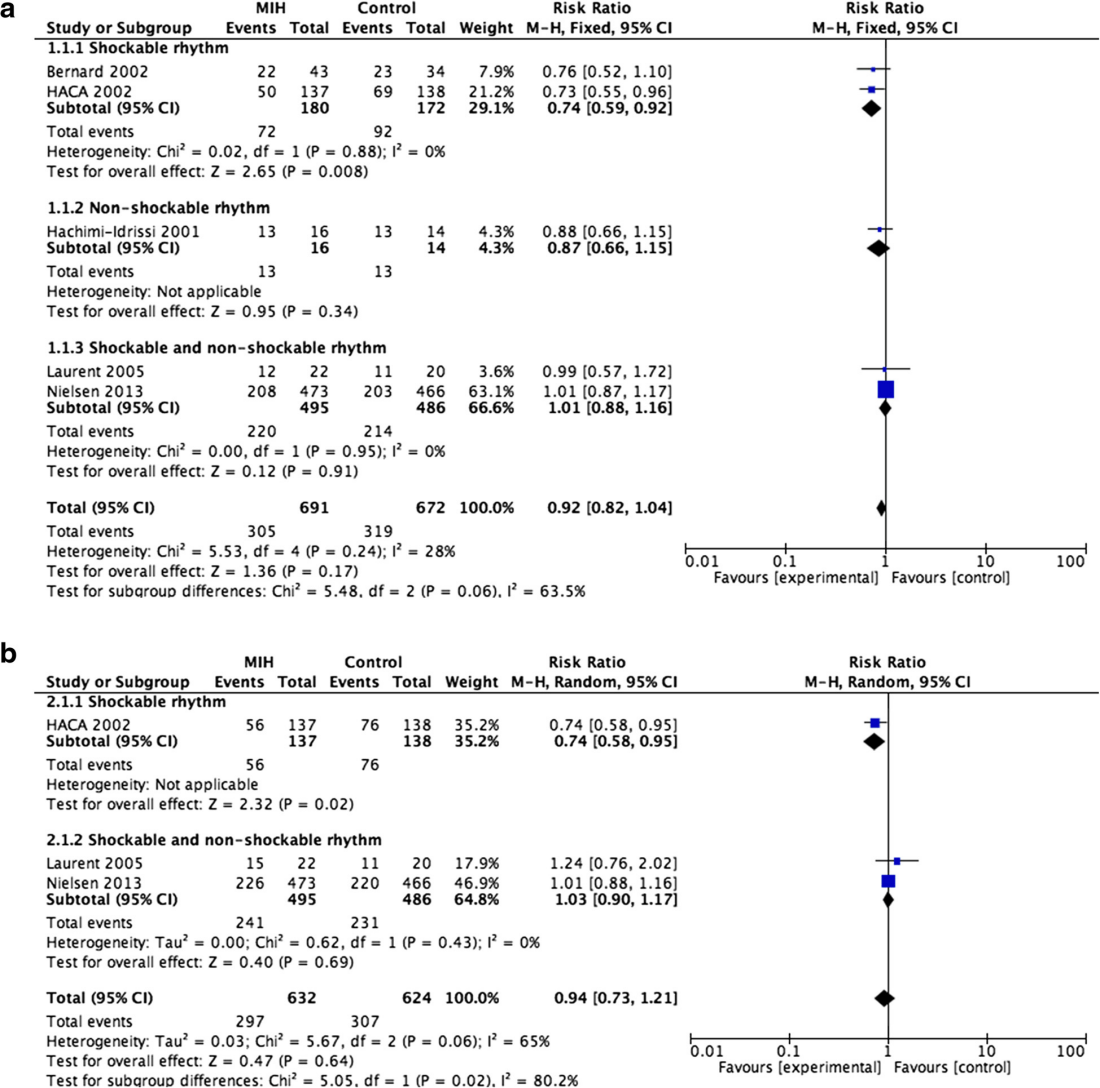
**a**. Forest plots of the effects of mild induced hypothermia on the mortality at hospital discharge of patients after cardiac arrest. **b**. Forest plots of the effects of mild induced hypothermia on the mortality at 6 months or 180 days of patients after cardiac arrest. *CI* Confidence interval, *HACA* Hypothermia After Cardiac Arrest, *I* ^*2*^ Percentage of total variation across studies from between-study heterogeneity rather than by chance, *M*-*H* Mantel-Haenszel, MIH mild induced hypothermia

Among the included studies, five studies of 1363 patients reported the mortality at hospital discharge and were included in the primary analysis. We detected no evidence of a publication bias after a funnel plot analysis (Additional file 2: Figure S1a). There was statistically insignificant heterogeneity (*p* = 0.24) and medium heterogeneity (I^2^ = 28 %) among all mortality at discharge analyses (Fig. 3a). The mortality rate was not significantly different between the MIH group and the control group (RR = 0.92; 95 % CI, 0.82–1.04; *p* = 0.17). However, a subgroup analysis showed that MIH could reduce the mortality of patients who had shockable rhythms and cardiac arrest (n = 352; RR = 0.74; 95 % CI, 0.59–0.92; *p* = 0.008). The RR of mortality for patients with non-shockable rhythms who received MIH versus those who did not (control group) was 0.87 (n = 30; 95 % CI, 0.66–1.15; *p* = 0.34).

Among the included studies, only three studies of 1256 patients reported the mortality at 6 months or 180 days. We detected no evidence of a publication bias after a funnel plot analysis (Additional file 2: Figure S1b), but there was significant heterogeneity (*p* = 0.06) and substantial heterogeneity (I^2^ = 65 %) among the trials (Fig. 3b). Mortality at 6 months or 180 days was not reduced in patients treated with MIH compared to control (RR = 0.94; 95 % CI, 0.73–1.21; *p* = 0.64). However, a subgroup analysis showed that MIH could reduce the mortality at 6 months or 180 days of patients who had shockable rhythms (n = 275; RR = 0.74; 95 % CI, 0.58–0.95; *p* = 0.02).

### Impact on neurological function

**Fig. 4:**
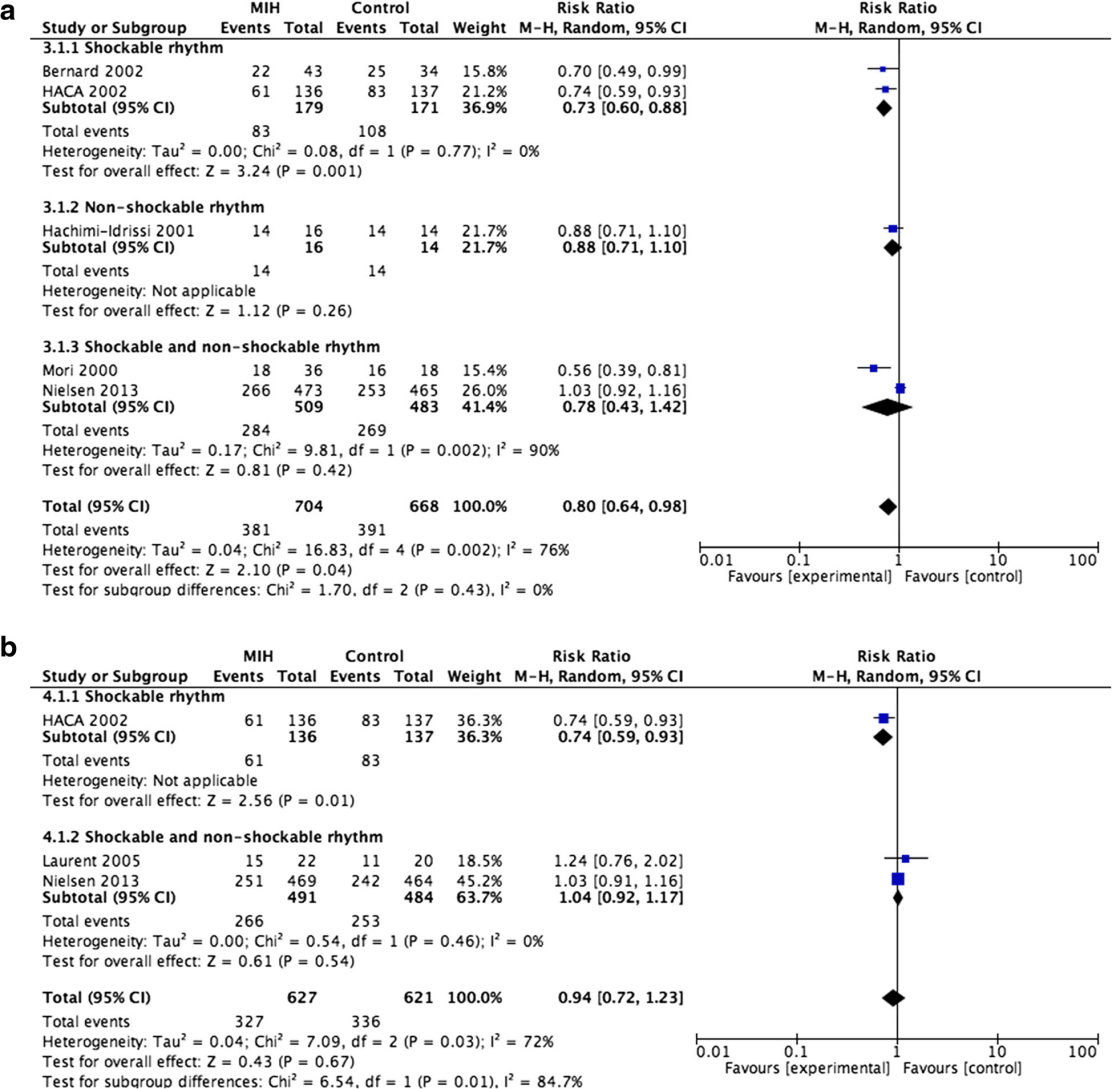
**a**. Forest plots of the effects of mild induced hypothermia on neurological function at hospital discharge in patients after cardiac arrest. **b**. Forest plots of the effects of mild induced hypothermia on neurological function at 6 months or 180 days in patients after cardiac arrest. *CI* Confidence interval, *HACA* Hypothermia After Cardiac Arrest, *I* ^*2*^ Percentage of total variation across studies from between-study heterogeneity rather than by chance, *M*-*H* Mantel-Haenszel, MIH mild induced hypothermia

The overall effect of MIH on neurological function at hospital discharge was estimated from five trials, which included a total of 1372 patients. No evidence of a publication bias was observed following a funnel plot assessment (Additional file 3: Figure S2a), but there was significant heterogeneity (*p* = 0.002) and substantial heterogeneity (I^2^ = 76 %) among the trials (Fig. 4a). Based on a random-effects model, we found that MIH promotes better outcomes of neurological function at hospital discharge (RR = 0.80; 95 % CI, 0.64–0.98; *p* = 0.04). Interestingly, similar to the effect of MIH on mortality, the subgroup analysis showed that MIH could also improve the neurological function of patients with shockable rhythms (n = 350; RR = 0.73; 95 % CI, 0.60–0.88; *p* = 0.001), but not those with non-shockable rhythms (n = 30; RR = 0.88; 95 % CI, 0.77–1.10; *p* = 0.26).

The overall effect of MIH on neurological function at 6 months or 180 days was estimated from only three trials of 1348 patients. No evidence of a publication bias was observed following a funnel plot assessment (Additional file 3: Figure S2b), but there was significant heterogeneity (*p* = 0.03) and substantial heterogeneity (I^2^ = 72 %) among the trials (Fig. 4b). MIH did not promote better outcomes of neurological function at 6 months or 180 days based on a random-effects model (RR = 0.94; 95 % CI, 0.72–1.23; *p* = 0.67). However, the subgroup analysis showed that MIH could also improve the neurological function of patients with shockable rhythms (n = 273; RR = 0.74; 95 % CI, 0.59–0.93; *p* = 0.01).

### Impact on complications

**Fig. 5:**
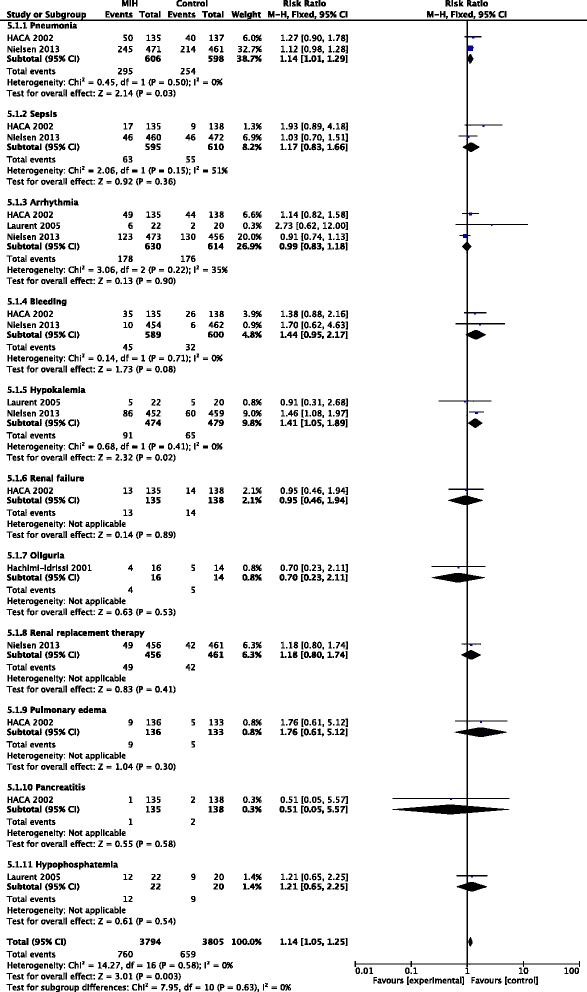
Forest plots of adverse events associated with mild induced hypothermia in patients after cardiac arrest. *CI* Confidence interval, *HACA* Hypothermia After Cardiac Arrest, *I* ^*2*^ Percentage of total variation across studies from between-study heterogeneity rather than by chance, *M*-*H* Mantel-Haenszel, MIH mild induced hypothermia

Five trials reported the adverse effects of MIH but only four of them were evaluated to estimate the overall effect of MIH on the incidence of complications (Fig. 5) because the trial of Bernard et al. [6] did not report the incidence of the complications. The complications associated with MIH that were reported in the trials included pneumonia, sepsis, and arrhythmia, among others. The incidence of complications in the MIH group was significantly higher than that in the control group (RR = 1.14; 95 % CI, 1.05–1.25; *p* = 0.003). No evidence of a publication bias was observed following a funnel plot assessment (Additional file 4: Figure S3).

### Risk of bias in included studies

**Fig. 6:**
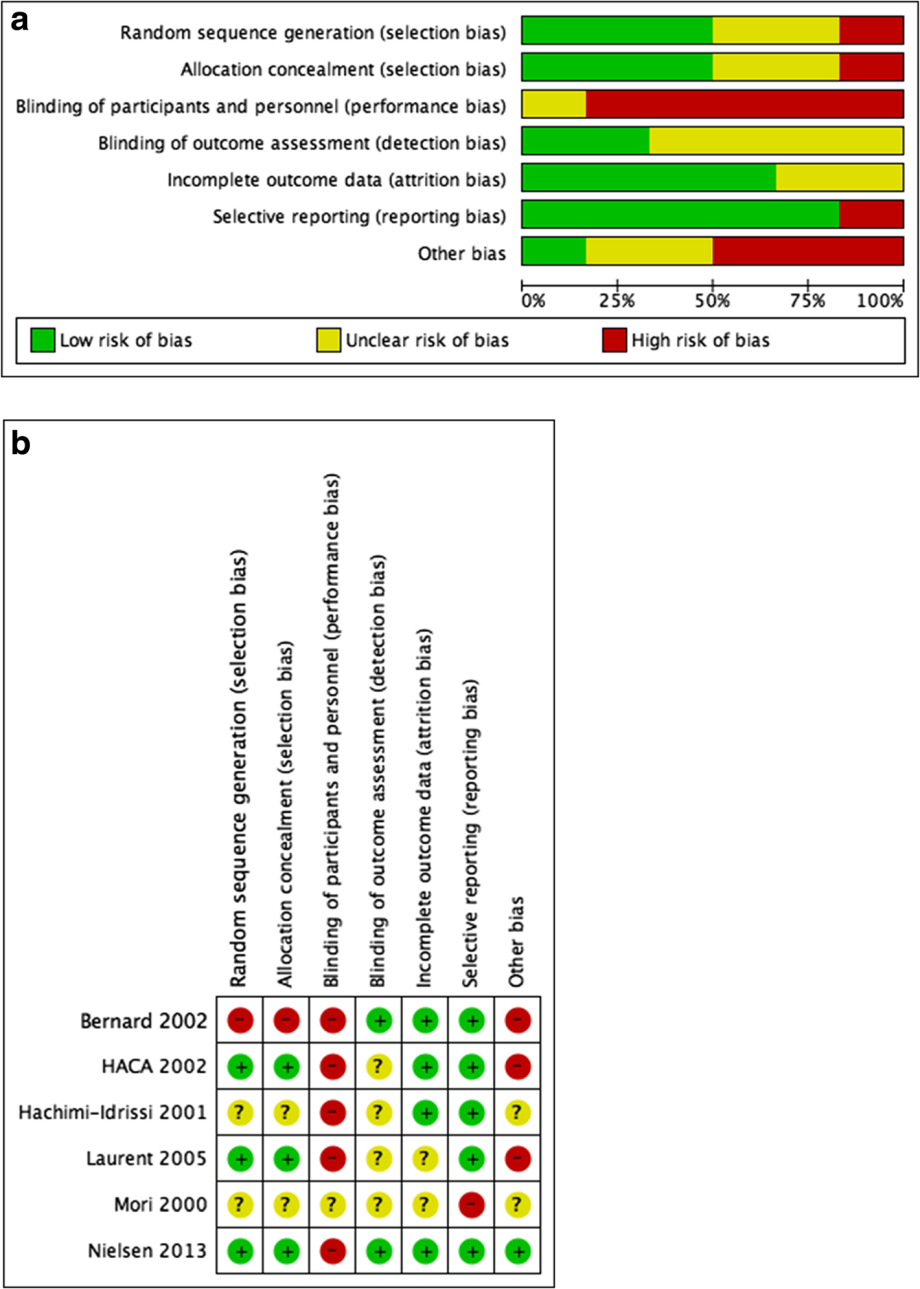
**a**. Risk of bias graph. Review authors’ judgements about each risk of bias item presented as percentages across all included studies. **b**. Risk of bias summary. Review authors’ judgements about each risk of bias item for each included study

We assessed each included trial by the mode of randomization, allocation concealment, level of blinding and loss to follow-up (Fig. 6).

### Summary of evidence according to GRADE

Randomised trials are rated high on the GRADE scale. There were variable risks of bias in all the trials, leading us to downgrade the quality of the evidence. Due to one trial [11], there was some inconsistency among the trials. One trial [7] included only less than 8 % of the screened patients and one trial [22] included only CA patients with pulseless electrical activity and asystole. Our application of GRADE methodology led us to conclude that the accumulated evidence is of low quality for mortality and neurological outcome. For a GRADE profile see Tables 2 and 3.

**Table 2:** Summary of findings for the main comparison

MIH compared to control for cardiac arrest patients						
Setting:						
Intervention: MIH						
Comparison: Control						
Patient or population: cardiac arrest patients						
Setting:						
Intervention: MIH						
Comparison: Control						
Outcomes	Anticipated absolute effects^a^ (95 % CI)		Relative effect (95 % CI)	No. of participants (studies)	Quality of the evidence (GRADE)	Comments
	Risk with control	Risk with MIH				
Mortality (follow-up 180 days or hospital discharge)	Study population		RR 0.94 (0.84 to 1.04)	1363 (5 RCTs)	⨁◯◯◯ VERY LOW^b c d^	
	510 per 1000	480 per 1000 (429 to 531)				
	Moderate					
	551 per 1000	518 per 1000 (463 to 573)				
Neurological outcome (follow-up 180 days or hospital discharge)	Study population		RR 0.83 (0.68 to 1.01)	1409 (6 RCTs)	⨁◯◯◯ VERY LOW^b c d^	
	569 per 1000	472 per 1000 (387 to 575)				
	Moderate					
	671 per 1000	557 per 1000 (456 to 677)				

*GRADE* Working Group grades of evidence: high quality—we are very confident that the true effect lies close to that of the estimate of the effect; moderate quality—we are moderately confident in the effect estimate (the true effect is likely to be close to the estimate of the effect, but there is a possibility that it is substantially different); low quality—our confidence in the effect estimate is limited (the true effect may be substantially different from the estimate of the effect); very low quality—we have very little confidence in the effect estimate (the true effect is likely to be substantially different from the estimate of effect).

^a^The risk in the intervention group (and its 95 % CI) is based on the assumed risk in the comparison group and the relative effect of the intervention (and its 95 % CI)

^b^All trials were with substantial risk of bias

^c^One trial accounted for the largest part among all the trials and probably contributed to the heterogeneity

^d^One trial included only less than 8 % of the screened patients. One trial included only cardiac arrest patients with pulseless electrical activity and asystole

*CI* Confidence interval, *MIH* Mild induced hypothermia, *RCT* Randomised controlled trial, *RR* Risk ratio

**Table 3:** GRADE profile for assessing quality of evidence for mild induced hypothermia after out-of-hospital cardiac arrest

Quality assessment							No. of patients		Effect		Quality	Importance
No. of studies	Study design	Risk of bias	Inconsistency	Indirectness	Imprecision	Other considerations	MIH	Control	Relative (95 % CI)	Absolute (95 % CI)		
Mortality (follow-up 180 days or hospital discharge)												
5	Randomised trials	Serious^a^	Serious^b^	Serious^c^	Not serious	None	332/691 (48.0 %)	343/672 (51.0 %)	RR 0.94 (0.84–1.04)	31 fewer per 1000 (from 20 more to 82 fewer)	⨁◯◯◯ VERY LOW^a b c^	CRITICAL
								55.1 %		33 fewer per 1000 (from 22 more to 88 fewer)		
Neurological outcome (follow-up 180 days or hospital discharge)												
6	Randomised trials	Serious^a^	Serious^b^	Serious^c^	Not serious	None	381/722 (52.8 %)	391/687 (56.9 %)	RR 0.83 (0.68–1.01)	97 fewer per 1000 (from 6 more to 182 fewer)	⨁◯◯◯ VERY LOW^a b c^	CRITICAL
								67.1 %		114 fewer per 1000 (from 7 more to 215 fewer)		

*GRADE* Working Group grades of evidence: high quality—we are very confident that the true effect lies close to that of the estimate of the effect; moderate quality—we are moderately confident in the effect estimate (the true effect is likely to be close to the estimate of the effect, but there is a possibility that it is substantially different); low quality—our confidence in the effect estimate is limited (the true effect may be substantially different from the estimate of the effect); very low quality—we have very little confidence in the effect estimate (the true effect is likely to be substantially different from the estimate of effect).

^a^All trials were with substantial risk of bias

^b^One trial accounted for the largest part among all the trials and probably contributed to the heterogeneity

^c^One trial included only less than 8 % of the screened patients. One trial included only cardiac arrest patients with pulseless electrical activity and asystole

*CI* Confidence interval, *MIH* Mild induced hypothermia, *RR* Risk ratio

### Sensitivity analysis

**Fig. 7:**
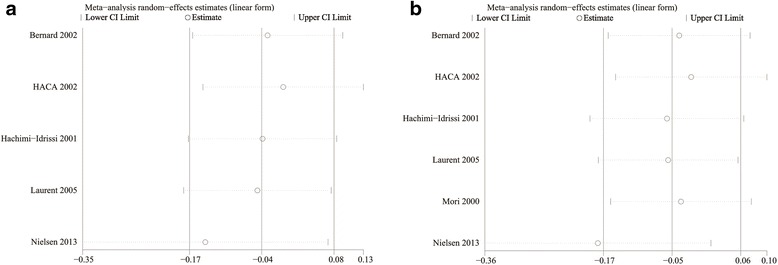
**a**. Sensitivity analysis of the effects of mild induced hypothermia on the mortality of patients after cardiac arrest for all included studies. **b**. Sensitivity analysis of the effects of mild induced hypothermia on the neurological function of patients after cardiac arrest for all included studies. *CI* Confidence interval, *HACA* Hypothermia After Cardiac Arrest

As shown in Fig. 7a and b, the study conducted by Nielsen et al. [11] was completely out of range of the others and probably contributed to the heterogeneity, indicating lack of reliability in our conclusions.

### Impact of MIH versus no target temperature on mortality and neurological function of CA patients

Among the included studies, four studies of 423 patients reported the mortality and four studies of 421 patients reported neurological function at 6 months and at hospital discharge. We detected no evidence of a publication bias after a funnel plot analysis (Additional file 5: Figure S4a and b). The mortality rate was significantly different between the MIH group and the no target temperature group (RR = 0.77; 95 % CI, 0.65–0.92; *p* = 0.003), and so was the neurological function between them (RR = 0.76; 95 % CI, 0.65–0.89; *p* < 0.001) (Additional file 6: Figure S5a and b).

### Impact of MIH versus TTM on mortality and neurological function of CA patients

Among the included studies, only two studies of 980 patients reported the mortality and three studies of 1029 patients reported neurological function at the end of trial and at hospital discharge. We detected no evidence of a publication bias after a funnel plot analysis, but there was significant heterogeneity (*p* = 0.005) and substantial heterogeneity (I^2^ = 81 %) among the trials in the effect on neurological function (Additional file 7: Figure S6a and b). The mortality rate was not significantly different between the MIH group and the TTM group (RR = 1.03; 95 % CI, 0.91–1.16; *p* = 0.67), and neither was the neurological function between them (RR = 0.89; 95 % CI, 0.59–1.35; *p* = 0.58) (Additional file 8: Figure S7a and b).

## Discussion

MIH is one of the most common treatments for patients who experience CA. However, whether it can improve the prognosis of patients with CA is still controversial. To further evaluate the effects of MIH compared to controls with temperature >34 °C in adult patients after CA on mortality and neurologic performance, a meta-analysis of RCTs was performed. The results suggest that: 1) MIH did not significantly decrease the mortality at hospital discharge or at 6 months or 180 days, but it did reduce the mortality of patients with shockable rhythms at hospital discharge and at 6 months or 180 days; 2) MIH can improve the outcome of neurological function at hospital discharge especially in those patients with shockable rhythm but not at 6 months or 180 days; 3) The incidence of complications in the MIH group was significantly higher than that in the control group; 4) All of the intervention effects above could not be confirmed or rejected by TSA.

The results of the meta-analysis were different from previous studies investigating possible beneficial effects of MIH after CA. To our knowledge, our study is the first meta-analysis that separated the patients into shockable and non-shockable rhythm due to the initial rhythms. Moreover, our study also included the study by Nielsen et al. [11] that has a very low risk of bias. In contrast to the last Cochrane reviews [12], we did not find a reduced mortality comparing MIH and TTM. Furthermore, Cochrane reviews [12] did not report any outcome at 6 months. In the meta-analysis by Nielsen et al. [10] in 2011, the data on main outcomes were also pooled together. In our meta-analysis, we used TSA to evaluate type I errors which result from an increased risk of random error and repeated significance testing.

Heterogeneity in the control group makes this meta-analysis different from others. It is very clear to demonstrate the overall effects of MIH but imprecise to state the influences of the exact range of temperature when mixing the controls with any body temperature >34 °C together. However, when separating the temperature control into no target temperature and TTM in the subgroup analysis, it shows that patients treated with MIH have a better outcome than patients treated with no target temperature. Because of the heterogeneous control in the meta-analysis, it does not mean that temperature does not need to be managed at all for CA patients. Current 2015 American Heart Association Guidelines Update for Cardiopulmonary Resuscitation and Emergency Cardiovascular Care recommend that all comatose adult patients with ROSC after CA should have TTM, with a target temperature between 32 °C and 36 °C selected and achieved, then maintained constantly for at least 24 hours [23]. Therefore, further trials are needed to elucidate the optimal target temperature of MIH after CA.

That MIH cannot improve the mortality of patients with CA may be due to the heterogeneity of patients. Whole-body low temperature treatment during resuscitation may interfere with organ function, and thus the benefits of MIH should be balanced with its side effects [24]. Moreover, large differences remain in patients with CA of different severity, and the potential pros and cons of temperature intervention are not equivalent in different patients. Recently, a large observational study has shown that hypothermia was independently associated with an improved outcome at hospital discharge in patients who presented with ventricular fibrillation/ventricular tachycardia but not in patients with non-shockable rhythms [25]. Therefore, if the time and degree of hypothermia could match the severity of the brain injury in patients with CA, these patients may also benefit from the MIH. However, these patients were not screened before each study, and a “one-size-fits-all” treatment approach makes it impossible to improve the outcomes of patients who require this treatment.

The selection of the temperature and implementation time of MIH may also affect the improvement of neurological function. The primary protective effect of MIH is to reduce brain metabolism of free radicals, to inhibit the release of excitatory amino acids, to attenuate the immune response in reperfusion and to inhibit apoptosis of brain cells [26]. Lopez-de-Sa and colleagues have suggested that a 32 °C cooling level may be associated with lower mortality and incidence of seizures compared with that of a 34 °C cooling level in patients who experienced an OHCA secondary to a shockable rhythm [27]. Moreover, in animal models of CA, the implementation of low temperature treatment (33 °C ± 1 °C in 4 hours after ROSC) can improve mortality and neurological function [28, 29]. In addition, it has been demonstrated that prehospital therapeutic hypothermia after CA can decrease temperature on hospital admission [30]. Therefore, a quicker induction of hypothermia may prevent a cascade effect of reperfusion injury, inflammatory attack and cell degeneration. However, current guidelines for CA do not recommend routine prehospital cooling with rapid infusion of cold intravenous fluids after ROSC [23]. MIH also interferes with numerous physiological and pathological processes; therefore, MIH might have unfavourable effects in patients who receive it and potentially put them at risk for adverse events.

Different cooling measurements may cause different complications and may reduce the efficacy of MIH. Surface and intravascular (invasive) cooling are the main measures for induction and maintenance of low body temperature. Compared with intravascular cooling, simple surface cooling seems to be associated with greater temperature fluctuations and more frequent overcooling, which may result in serious complications [31]. Nonetheless, intravascular cooling and surface cooling with automatic feedback systems allow for a more stable control of the temperature [32, 33]. Therefore, although no significant differences were observed in the complications between the low temperature and the normal temperature groups in patients with CA [34], the appropriate cooling measures could effectively prevent potential complications associated with MIH. Many cooling methods currently exist in clinical practice, and the identification of the most effective and safe methods to induce hypothermia need to be confirmed by further research.

The avoidance of post-hypothermia fever (PHF) can effectively improve the prognosis of patients with CA. Two large RCTs were included in our meta-analysis, namely the study by Nielsen et al. [11] and the Hypothermia After Cardiac Arrest (HACA) study [7]. After a comparison of the two studies, the mortality of OHCA patients in Nielsen et al.’s study was lower than that of patients in the HACA study. This is likely related to control of the body temperature after MIH. Moreover, that no differences were observed in the outcomes of the two groups of patients in Nielsen et al.’s study seems to be associated with a prevention of PHF. In addition, in older trials of MIH, many patients in the “normothermia” group actually became hyperthermic, which is deleterious for prognosis and recovery of neurological function in patients with CA. Bro-Jeppesen and colleagues have shown that a PHF ≥38.5 °C was associated with an increase in 30-day mortality, even after controlling for potential confounding factors [35]. Current guidelines also show that actively preventing fever in comatose patients after temperature management is reasonable [23]. Therefore, effectively avoiding PHF may benefit patients with CA.

It is worth mentioning that there is lack of firm evidence for a beneficial effect and an insufficient information size to reject the anticipated intervention effect. Thus, the question whether MIH is beneficial, neutral or harmful, for adult OHCA patients still needs an answer.

## Conclusions

In summary, this meta-analysis of the available RCTs showed that MIH does not improve in- hospital, 6-month or 180-day mortality in adult patients with CA. However, it is too early to completely negate the therapeutic effects of MIH in patients with CA. Future studies should pay particular attention to the temperature and the timing of MIH, selection of patients, efficiency and security of cooling measures and the avoidance of PHF. Much more research is needed to optimise the strategy of MIH.

## Key messages

• Mild induced hypothermia is believed to reduce mortality and neurological impairment after out-of-hospital cardiac arrest. However, a recently published trial did not provide evidence supporting this hypothesis.

• Our study-level meta-analysis shows that treatment with mild induced hypothermia does not appear to improve the short-term or long-term mortality of adult patients with cardiac arrest.

• Mild induced hypothermia may have a possible beneficial effect for patients with shockable rhythms both in mortality and neurological function.

• Although mild induced hypothermia may result in some adverse events, it helped lead to better outcomes regarding neurological function in patients with cardiac arrest, especially those with shockable rhythms. Large-scale ongoing trials addressing this question may provide data better applicable to clinical practice.

## Additional files

Additional file 1: Table S1. Search strategy for the Cochrane database. Table S2 Search strategy for other databases. (DOCX 63 kb) Additional file 2: Figure S1. a. Funnel plots of the effects of mild induced hypothermia on the mortality at hospital discharge of patients after cardiac arrest. b. Funnel plots of the effects of mild induced hypothermia on the mortality at 6 months or 180 days of patients after cardiac arrest. (TIF 354 kb) Additional file 3: Figure S2. a. Funnel plots of the effects of mild induced hypothermia on neurological function at hospital discharge in patients after cardiac arrest. b. Funnel plots of the effects of mild induced hypothermia on neurological function at 6 months or 180 days of patients after cardiac arrest. (TIF 344 kb) Additional file 4: Figure S3. Funnel plots of adverse events associated with mild induced hypothermia in patients after cardiac arrest. (TIF 890 kb) Additional file 5: Figure S4. a. Funnel plots of the effects of MIH compared with no target temperature on the mortality of patients after cardiac arrest. b. Funnel plots of the effects of MIH compared with no target temperature on neurological function of patients after cardiac arrest. (TIF 341 kb) Additional file 6: Figure S5. a. Forest plots of the effects of MIH compared with no target temperature on the mortality of patients after cardiac arrest. b. Forest plots of the effects of MIH compared with no target temperature on neurological function of patients after cardiac arrest. (TIF 2865 kb) Additional file 7: Figure S6. a. Funnel plots of the effects of MIH compared with target temperature management on the mortality of patients after cardiac arrest. b. Funnel plots of the effects of MIH compared with target temperature management on neurological function of patients after cardiac arrest. (TIF 340 kb) Additional file 8: Figure S7. a. Forest plots of the effects of MIH compared with target temperature management on the mortality of patients after cardiac arrest. b. Forest plots of the effects of MIH compared with target temperature management on neurological function of patients after cardiac arrest. (TIF 2174 kb)

## Abbreviations

CA: Cardiac arrest
CI: Confidence interval
CPC: Cerebral performance category
CRRT: Continuous renal replacement therapy
DIS: Diversity-adjusted information size
HACA: Hypothermia After Cardiac Arrest
IHCA: In-hospital cardiac arrest
M-H: Mantel-Haenszel
MIH: Mild induced hypothermia
OHCA: Out-of-hospital cardiac arrest
PHF: Post-hypothermia fever
RCT: Randomised controlled trial
ROSC: Return of spontaneous circulation
RR: Risk ratio
RRR: Relative risk reduction
TSA: Trial sequential analysis
TTM: Target temperature management.
