# Supplementary material for: The effect of mild induced hypothermia on outcomes of patients after cardiac arrest: a systematic review and meta-analysis of randomised controlled trials
Source: Crit Care. 2015 Dec 1;19:417. doi: 10.1186/s13054-015-1133-0 (PMC4665688; doi:10.1186/s13054-015-1133-0)
Supplement: Additional file 1: Table S1. — Search strategy for the Cochrane database. Table S2 Search strategy for other databases. (DOCX 63 kb) [file 13054_2015_1133_MOESM1_ESM.docx]

**Appendix Table 1** Search strategy for the Cochrane database

| #1 MeSH descriptor Resuscitation explode all trees  #2 MeSH descriptor Cardiopulmonary Resuscitation explode all trees  #3 MeSH descriptor Resuscitation Orders explode all trees  #4 MeSH descriptor Heart Arrest explode all trees  #5 MeSH descriptor Heart Massage explode all trees  #6 ((cardio?pulmonary or order*) near2 resuscitation):ti,ab  #7 reanimation:ti,ab  #8 ((circulatory or circulation or cardiac) near arrest):ti,ab or heart standstill:ti,ab  #9 (#1 OR #2 OR #3 OR #4 OR #5 OR #6 OR #7 OR #8)  #10 MeSH descriptor Cryotherapy explode all trees  #11 MeSH descriptor Hypothermia explode all trees  #12 MeSH descriptor Hypothermia, Induced explode all trees  #13 ((resuscitative or therapeutic or artificial or induced or extracorporeal) near hypothermia)  #14 artificial hibernation or body cooling or refrigeration anesthesia or body temperature:ti,ab or refrigeration:ti,ab  #15 (#10 OR #11 OR #12 OR #13 OR #14)  #16 (#9 AND #15) |
| --- |

**Appendix Table 2** Search strategy for other databases

| ① Randomised clinical trials |
| --- |
| ② Cardiac arrest OR out-of-hospital cardiac arrest OR OHCA OR in-hospital cardiac arrest OR IHCA OR circulatory arrest OR heart stop OR resuscitation OR cardiopulmonary OR CPR |
| ③ Hypothermia OR therapeutic hypothermia OR induced hypothermia OR mild hypothermia OR temperature control OR temperature management OR thermoregulatory management OR thermoregulatory control OR chill therapy OR cooling OR body temperature |
| ④ Neurological function OR neurological recovery OR neurological outcome OR cerebral performance category OR CPC OR survival OR outcome OR temperature OR ischemia OR brain ischemia OR cerebral ischemia OR global ischemia resuscitation |
| ⑤ Humans |
| ① AND ② AND (③ OR ④) AND ⑤ |

IHCA: in-hospital cardiac arrest; OHCA: out-of-hospital cardiac arrest
